# Development of a risk score for scoliosis in children with cerebral palsy

**DOI:** 10.1080/17453674.2020.1711621

**Published:** 2020-01-13

**Authors:** Katina Pettersson, Philippe Wagner, Elisabet Rodby-Bousquet

**Affiliations:** a Department of Clinical Sciences, Lund University, Orthopedics, Lund, Sweden;; b Centre for Clinical Research, Region Västmanland—Uppsala University, Västerås, Sweden

## Abstract

Background and purpose — Children and young adults with cerebral palsy (CP) have an increased risk of developing scoliosis, with a prevalence ranging from 11% to 29%. Information on risk factors for the emergence and progression of scoliosis is inconclusive. This study aimed to develop a risk score based on 5-year-old children with CP to predict the risk of scoliosis before the age of 16.

Patients and methods — This prospective registry study included 654 children with CP in Sweden born in 2000 to 2003 and registered with the Swedish CP follow-up program (CPUP) at the age of 5 years, including all Gross Motor Function Classification System (GMFCS) levels. 92 children developed a scoliosis before the age of 16 years. Univariable and multivariable logistic regressions were used to analyze 8 potential predictors for scoliosis: GMFCS, sex, spastic subtype, epilepsy, hip surgery, migration percentage, and limited hip or knee extension.

Results — 4 predictors for scoliosis remained significant after analyses: female sex, GMFCS levels IV and V, epilepsy, and limited knee extension, and a risk score was constructed based on these factors. The predictive ability of the risk score was high, with an area under the receiver operating characteristics curve of 0.87 (95% CI 0.84–0.91).

Interpretation — The risk score shows high discriminatory ability for differentiating between individuals at high and low risk for development of scoliosis before the age of 16. It may be useful when considering interventions to prevent or predict severe scoliosis in young children with CP.

Children and adults with cerebral palsy (CP) have a high risk of developing scoliosis (Saito et al. 1998, Hägglund et al. 2018a). The risk for neuromuscular scoliosis also increases with age (Persson-Bunke et al. 2012, Hägglund et al. 2018a) and early onset of scoliosis has been identified as a predictor for severe scoliosis (Saito et al. 1998, Gu et al. 2011, Persson-Bunke et al. 2012, Yoshida et al. 2018). To date, information on the risk factors for emergence and progression of scoliosis in children with CP is inconclusive (Loeters et al. 2010).

The Gross Motor Function Classification System (GMFCS) levels III, IV, and V have been identified as risk factors for scoliosis in children with CP (Loeters et al. 2010, Hägglund et al. 2018a). New findings suggest that girls with CP have a higher risk than boys of developing scoliosis (Bertoncelli et al. 2017, Hägglund et al. 2018a, Pettersson et al. 2019), and that epilepsy is another independent risk factor (Bertoncelli et al. 2017). Lateral displacement of the hips, hip dislocations, and previous hip surgery are sometimes associated with neuromuscular scoliosis (Persson-Bunke et al. 2006, Bertoncelli et al. 2017, Hägglund et al. 2018b), whereas successful hip surveillance leading to a reduced number of dislocated hips results in a lower proportion of scoliosis (Hägglund et al. 2014). In addition, limited hip or knee extension is highly associated with scoliosis, windswept hips, and postural asymmetries in adults with CP (Rodby-Bousquet et al. 2013, Ágústsson et al. 2018).

We developed a risk score to predict the individual risk for a 5-year-old child with CP to develop a severe scoliosis before the age of 16 years. This individual risk score can prompt clinicians to initiate and implement preventive interventions and strategies at an early stage and hopefully reduce the risk of scoliosis.

## Patients and methods

This prospective registry study was based on data from the combined Swedish Cerebral Palsy Follow up-program and National quality registry (CPUP) (Hägglund et al. 2014, Alriksson-Schmidt et al. 2017). We included all children with CP in Sweden born in 2000 to 2003 who were reported to the registry at 2 time points: at 5 years and before the age of 16 years. Over 95% of all children with CP in Sweden participate in CPUP (Westbom et al. 2007). Children are assessed clinically twice a year until they are 6 years of age, and then once a year (Alriksson-Schmidt et al. 2014). CP diagnosis and neurological subtype are classified from the age of 4 years. Exclusion and inclusion criteria are consistent with those of the Surveillance of Cerebral Palsy network in Europe (SCPE) (2000).

Scoliosis was defined as either having: (1) a radiographically measured Cobb angle of at least 40° (Persson-Bunke et al. 2012, Hägglund et al. 2018a); (2) a spinal fusion because of scoliosis; or (3) a severe scoliosis at clinical examination (Persson-Bunke et al. 2015) before the age of 16 years. In CPUP, clinical assessment of the spine is used as a screening tool to identify children in need of further radiographic examination. The clinical examination is performed with the child in a sitting position, both upright and forward-bending. A pronounced curve preventing the child from attaining an upright position without external support is rated as severe scoliosis and treated as scoliosis in this study. This standardized clinical spinal assessment has high interrater reliability, sensitivity, specificity, and criterion-related validity compared with a radiographically measured Cobb angle (Persson-Bunke et al. 2015). Mild or moderate curves do not exceed 25 degrees of Cobb angle (Persson-Bunke et al. 2015), and in this study were treated as having no scoliosis.

Timepoint 1 used data from the assessment performed closest to the 5th birthday of each child, and timepoint 2 used the latest assessment before 16 years of age. Based on previous findings the following 8 variables were analyzed as potential predictors of scoliosis: GMFCS levels IV and V, female sex, spastic subtype, epilepsy, hip surgery, migration percentage (MP) > 40%, and limited hip or knee extension. Gross motor function was classified using the expanded and revised version of the GMFCS, levels I to V (Palisano et al. 2008). We grouped and used GMFCS levels I to III (higher motor function) as the reference category to compare each GMFCS level, IV and V (lower motor function). Male was used as the reference category for sex (Bertoncelli et al. 2017, Hägglund et al. 2018a, Pettersson et al. 2019). Epilepsy was reported as Yes or No, with no epilepsy as the reference category (Bertoncelli et al. 2017). Neurological subtype was classified as spastic CP (spastic unilateral and bilateral CP), versus non-spastic CP (ataxic, dyskinetic, mixed type), which was used as the reference category (Bertoncelli et al. 2017, 2018). All types of hip surgery (including femur osteotomy, pelvic osteotomy, and adductor psoas tenotomy) were grouped as hip surgery, with no surgery used as the reference category (Bertoncelli et al. 2017). Lateral displacement/migration of the hip joint was measured using MP (Reimers 1980). The value for the worst side was used. We defined lateral migration as an MP > 40% (Hägglund et al. 2007, Hermanson et al. 2015), using MP ≤ 40% as the reference category. Passive range of motion (ROM) for hip and knee extension was measured by goniometer in a standardized position (www.cpup.se), and the value for the worst side was used for all analyses. Data were dichotomized into either full hip or knee extension or limited hip or knee extension (–5° or less), and the former was used as the reference category.

### Statistics

We analyzed the risk of developing scoliosis after 5 years of age and before the age of 16, using predictors measured at the age of 5 years. The odds ratio (OR) with 95% confidence interval (CI) for scoliosis was calculated using logistic regression for the following variables: GMFCS, sex, spastic CP, epilepsy, hip surgery, MP, passive hip extension, and knee extension. The first step in the analysis was to calculate ORs for each variable using univariable logistic regression. The next step was multivariable logistic regression analysis using a stepwise backward elimination process, whereby 1 explanatory variable (that with the highest nonsignificant p-value) at a time was removed from the regression model. This step was repeated until only significant variables remained in the model.

A risk score was constructed using the remaining variables as independent, significant predictors of scoliosis. A p-value of less than 0.05 was considered statistically significant. This risk score was then evaluated using the area under the receiver operating characteristics (ROC) curve (AUC). The AUC can be interpreted as the probability that a randomly selected child with scoliosis has a higher predicted risk of severe scoliosis before the age of 16 than a randomly chosen individual without scoliosis. An AUC value of 1 is considered perfect and a value of 0.5 no better than chance (Steyerberg 2019). For these statistical analyses, IBM SPSS Statistics v24 (IBM Corp, Armonk, NY, USA) was used.

The risk score AUC was additionally validated using 10-fold cross validation. The risk score development process was also validated using a different predictor selection approach, L1-penalized logistic regression (Tibshirani 1996, Zou and Hastie 2005). The latter analyses were performed using R (R Core Team 2019, R Foundation for Statistical Computing, Vienna, Austria).

### Ethics, funding, and potential conflicts of interest

The study was approved by the Medical Research Ethics Committee at Lund University (383/2007, 443-99), and permission was obtained to extract data from the CPUP registry. The study was funded by the Norrbacka-Eugenia Foundation, Region Västmanland, Promobilia, Stiftelsen för bistånd åt rörelsehindrade i Skåne and Forte. The funding sources had no decision-making role or influence on the study design, data collection, data analysis, data interpretation, or writing of the report. The authors declare that they have no conflicts of interest.

## Results

The risk score was based on data of all 654 children with CP born in Sweden from 2000 to 2003, with data reported closest to 5 years and before 16 years of age (Table 1). Their ages ranged from 4.0 to 5.9 years. We identified 92/654 (14%) individuals who met our criteria for severe scoliosis before the age of 16. Of these children, 59 had undergone spinal fusion for scoliosis, a further 6 had a reported Cobb angle of at least 40°, and 27 had severe scoliosis identified on clinical examination.

**Table 1.** Demographic distribution for included variables. Values are frequency (%)

Variable/category	n = 654
GMFCS ^a^	
I	264 (40)
II	117 (18)
III	53 (8.1)
IV	115 (18)
V	105 (16)
Sex	
Male	372 (57)
Female	282 (43)
CP ^b^ subtype	
Spastic unilateral	268 (41)
Spastic bilateral	216 (33)
Ataxic	41 (6.3)
Dyskinetic	93 (14)
Mixed type	22 (3.4)
Missing	14 (2.1)
Birthyear	
2000	113 (17)
2001	144 (22)
2002	191 (29)
2003	206 (32)
Epilepsy, yes	222 (34)
Hip surgery **^c^**	53 (8.1)
Worst side	
MP ^d^ 41–100%	45 (6.9)
Limited hip extension ≤ –5є	35 (5.4)
Limited knee extension ≤ –5є	91 (14)

**^a^**GMFCS = Gross Motor Function Classification System.

**^b^**CP = cerebral palsy.

**^c^**Femur-, pelvic osteotomy, adductor psoas tenotomy.

**^d^**MP = migration percentage.

**Figure 1.**
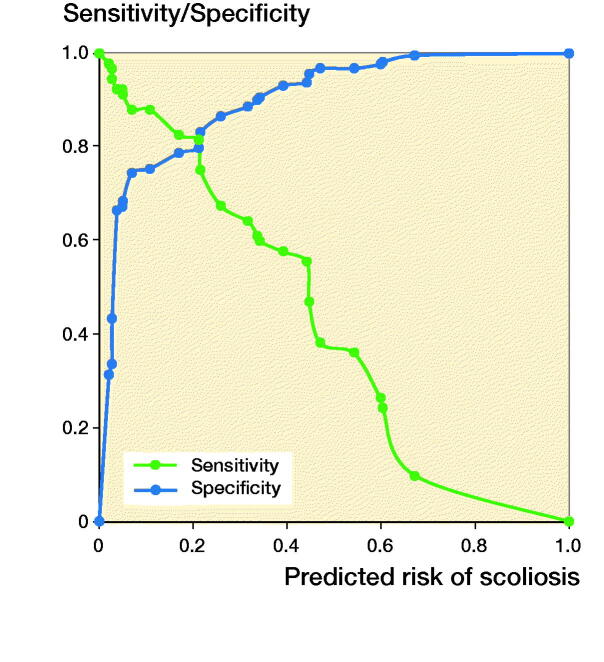
Graph showing the proportion of children with scoliosis correctly predicted to develop scoliosis before the age of 16 years (the sensitivity) and the proportion of children without scoliosis correctly predicted not to develop scoliosis before the age of 16 years (the specificity) for choice of a cutoff to indicate a high-risk individual.

Of the initial 8 possible predictors at the age of 5 years, 4 remained significantly associated with the development of severe scoliosis before the age of 16 years: female sex, GMFCS levels IV and V, epilepsy, and limited knee extension (Table 2). For the population frequencies of the 8 variables included, see Table 1. The sensitivity and (1 – specificity) of the risk score are shown in Figure 1. The sensitivity is the proportion of children with scoliosis correctly predicted to develop severe scoliosis before the age of 16 years. (1 – specificity indicates the proportion of those without scoliosis who were correctly predicted not to develop scoliosis before 16 years of age.) The discriminatory accuracy of the risk score was high, with an AUC = 0.87 (CI 0.84–0.91), indicating a strong ability to differentiate between high- and low-risk individuals. The AUC remained high after cross-validation, AUC = 0.866. The equation for calculating the risk score was determined to be:

Risk Score = –4.219 + 0.655·sex + 2.288·GMFCSIV + 3.366·GMFCSV + 0.622·epilepsy + 0.614·limited knee extension.

**Table 2.** Odds ratios (OR) with 95% confidence intervals (CI) for possible predictors of scoliosis measured at the age of 5 (steps 1 and 2) ^a^

Risk factors	Step 1 Univariable analysis OR (95% CI)	Step 2 Multivariable analysis, first regression OR (95% CI)	Step 3 Multivariable analysis, last regression OR (95% CI)	Step 4 L1-penalized regression OR
GMFCS I–III	Ref.	Ref.	Ref.	
GMFCS IV	12 (5.6–25)	10 (4.2–24)	9.9 (4.6–21)	8.4
GMFCS V	41 (20–83)	25 (9.5–65)	29 (14–61)	21
Female	1.7 (1.1–2.6)	1.9 (1.1–3.4)	1.9 (1.1–3.3)	1.8
Epilepsy	3.9 (2.5–6.2)	1.6 (0.9–2.9)	1.9 (1.1–3.2)	1.6
Knee extension ≤ –5°	4.3 (2.6–7.1)	1.9 (1.0–3.7)	1.9 (1.0–3.4)	1.8
Hip extension ≤ –5°	3.5 (1.7–7.3)	1.2 (0.5–3.0)		1.2
Spastic CP	0.4 (0.3–0.7)	1.3 (0.7–2.4)		1.2
Hip surgery	7.8 (4.3–14)	1.3 (0.6–2.9)		1.3
MP > 40%	5.9 (3.1–11)	1.3 (0.6–3.0)		1.3

**^a^** Step 3 shows the significant factors remaining after the stepwise procedure, which predict the development of scoliosis before the age of 16 years for children with cerebral palsy (CP).

Step 4 shows the ORs from the sensitivity analysis using L1-penalized regression.

Spastic CP includes uni- and bilateral spasticity.

For CP, GMFCS, Hip surgery, and MP, see Table 1

Sex is a dichotomous indicator variable that took a value of 1 for females and 0 for males. GMFCS IV is also a dichotomous indicator variable that took a value of 1 when the individual had GMFCS level IV, and 0 otherwise. GMFCS V is a corresponding indicator. Epilepsy took a value of 1 when epilepsy was present and 0 when it was not. Limited knee extension corresponded to the value on the worst side when both sides were measured and took the value of 1 when the limited knee extension was –5° or less, and 0 when the individual had full knee extension. The risk score can be translated into the risk of developing scoliosis using Table 3.

**Table 3.** Risk of developing scoliosis before the age of 16, corresponding to each risk score level

Risk score	Risk of scoliosis (%)
< –2.2	0 to 10
–2.2 to –1.4	10 to 20
–1.3 to –0.85	20 to 30
–0.85 to –0.41	30 to 40
–0.41 to 0	40 to 50
0 to 0.41	50 to 60
0.41 to 0.85	60 to 70
0.85 to 1.4	70 to 80
1.4 to 2.2	80 to 90
> 2.2	90 to 100

As an example, a female child at GMFCS level V, with epilepsy and limited ROM for knee extension, will have a risk score of 1.04 and a 70% to 80% risk of developing severe scoliosis before the age of 16.

The sensitivity analysis results are presented in Table 2 in the far-right column. AUC for the resulting risk score was marginally worse than the original risk score, with a cross-validated AUC of 0.85.

## Discussion

We developed a risk score based on the following risk factors assessed at the age of 5 years: female sex, GMFCS levels IV and V, epilepsy, and having limited knee extension in 654 children with CP in Sweden born in 2000 to 2003. These were identified as independent predictors for the development of scoliosis before the age of 16 years. The AUC of the resulting risk score was 0.87 (CI 0.84–0.91), indicating a high accuracy in differentiating between high- and low-risk individuals. The AUC remained at this level after cross-validation, showing that its high value was not due to overfitting, and may generalize to other populations. However, true external validity is yet to be verified in additional CP populations in future studies (Steyerberg and Harrell 2016). The sensitivity analysis further showed that our method had satisfactory performance in terms of selecting suitable predictors, at least compared with another popular approach, L1-penalized logistic regression (Ranstam and Cook 2018). To our knowledge, this is the first study creating a risk score for development of severe scoliosis based on predictors identified in 5-year old children with CP.

Scoliosis is usually defined as a radiographically measured lateral spinal curvature of at least 10° (Cobb 1948, Roberts and Tsirikos 2016), and a Cobb angle of ≥ 40° has been suggested as a cut-off for severe scoliosis when considering surgical interventions (Saito et al. 1998, Persson-Bunke et al. 2012, Hägglund et al. 2018a). But at present there are no internationally agreed criteria for the recommendation of spine surgery (Toovey et al. 2017). Scoliosis can also be identified at clinical examination. Clinically defined moderate or severe scoliosis show a sensitivity of 75% and a specificity of 96% compared with radiographic Cobb angle (Persson-Bunke et al. 2015). Even though scoliosis usually develops after the age of 8 years (Persson-Bunke et al. 2012), some children may start to develop scoliosis from the age of 5 years (Hägglund et al. 2018a). The most important and rapid growth spurt in children generally occurs from 11 to 14 years of age (Negrini et al. 2018). We therefore decided to identify potential variables at the age of 5 years to predict the risk for development of scoliosis before the age of 16 years.

Our results are consistent with previous findings (Persson-Bunke et al. 2012, Hägglund et al. 2018a) in identifying GMFCS as a strong predictor of scoliosis, with an OR of 9.9 for GMFCS level IV up to 29 for GMFCS level V. When only GMFCS was included in the ROC analysis, the AUC was 0.85 (CI 0.81–0.89). We included children at all GMFCS levels, thereby allowing comparisons between those with higher gross motor function (GMFCS levels I to III) and those with lower gross motor function (GMFCS levels IV and V). Notably, those at GMFCS levels I and II have a low incidence of scoliosis (Hägglund et al. 2018a). We found an increased risk for scoliosis in girls, with an OR of 1.9 for girls compared with boys, which confirms findings from recent studies (Bertoncelli et al. 2017, Hägglund et al. 2018a, Pettersson et al. 2019). Our study also confirmed the previous observation that epilepsy is a predictor of scoliosis (Bertoncelli et al. 2017), even after adjustment for other variables (Table 2). In supine lying, limited knee or hip extension may cause the legs to tilt to one side, forcing the pelvis and trunk into rotation. This deviation could be reinforced by time and gravity and is likely one of the reasons why limited knee and hip extension increases the probability for scoliosis and windswept hips in adults with CP (Ágústsson et al. 2018). With previous findings in mind (Rodby-Bousquet et al. 2013, Ágústsson et al. 2018, Cloodt et al. 2018), it is not surprising that limited knee extension contributes to the risk of developing scoliosis. Limited hip and knee extension may co-occur (Ágústsson et al. 2018), which might explain why limited hip extension did not remain significant in the multivariate regression analyses. Because knee contracture is one of the most common types of contracture (Rodby-Bousquet et al. 2013, Ágústsson et al. 2018, Cloodt et al. 2018), this reinforces the importance of monitoring range of motion and posture from an early age, and promptly addressing contractures and postural asymmetries.

The ROC curve (Figure 1) shows consecutive cutoffs for the predicted risk of scoliosis, and shows the possible cutoffs for classifying individuals as high- or low-risk (Steyerberg 2019). For a clinician to make a reasonable assessment of the risk of scoliosis, it would be advisable to base the decision regarding cutoffs on the nature of the treatment option being considered (Hermanson et al. 2015). For minor interventions like spinal orthosis, one should try to capture as many children as possible so as not to risk missing someone (high sensitivity). However, for major interventions like surgery, it is important to select a more conservative cutoff that includes only high-risk individuals, to prevent unnecessary interventions that in turn can have a negative impact on quality of life (high specificity). In general, spinal surgery is not performed before severe scoliosis is already present. However, our risk score may identify children who need close clinical and radiographic surveillance of their spine.

Most of the children included in this study have been followed regularly and received early interventions such as hip surgery in accordance with CPUP’s protocol and guidelines, meaning that by using a preventive follow-up program (that might include early corrective interventions), it can give protective effects of both hips and spines (Elkamil et al. 2011, Hägglund et al. 2014). This way of implementing early interventions may differ from other countries without follow-up programs. The chosen definition of limited knee extension with a cutoff value of –5° or more affected the observed frequency of knee contracture. A higher cutoff value, for example –10°, would have halved the prevalence from 14% to 7%. There might be other predictors that were not identified or included in this study. A strength of the study is that the risk score is based on the total population of children with CP who have been followed in a standardized way.

Scoliosis, once established, is usually a lifelong condition for individuals with CP (Hägglund et al. 2018a), in contrast to idiopathic scoliosis where the risk of further progression is much lower after spinal growth is complete (Negrini et al. 2018). It has been shown that young adults with CP at GMFCS levels IV and V have a 50% increased risk of moderate or severe scoliosis by the age of 18. By 20 years of age, 75% of adults at GMFCS level V have a Cobb angle ≥ 40° (Persson-Bunke et al. 2012, Hägglund et al. 2018a). This further reinforces the importance of early identification of children with CP at risk of developing scoliosis.

Based on this population of children with CP, we conclude that the presence of the following 4 predictors at the age of 5 years increases the probability of severe scoliosis before the age of 16 years: female sex, GMFCS levels IV and V, epilepsy, and having limited knee extension. The risk score has a high AUC and discriminatory accuracy for differentiating between children with CP and it is hoped will provide clinicians with early insight regarding a child’s risk of developing scoliosis and allow them to differentiate between high-risk and low-risk individuals.
